# Stability and Failure of Thin-Walled Composite Plate Elements with Asymmetric Configurations

**DOI:** 10.3390/ma17091943

**Published:** 2024-04-23

**Authors:** Katarzyna Falkowicz

**Affiliations:** Faculty of Mechanical Engineering, Lublin University of Technology, Nadbystrzycka 38, 20-618 Lublin, Poland; k.falkowicz@pollub.pl; Tel.: +48-81-538-4204

**Keywords:** FEM analysis, plate elements, thin-walled structures, failure analysis, failure of composites, critical and post-critical states

## Abstract

In the present study, the stability and failure phenomena of thin-walled constructions subjected to axial compression, featuring a central cut-out, and constructed from composite materials were explored. These constructions were fabricated from a carbon–epoxy composite using the autoclave method. The research encompassed experimental assessments on actual specimens alongside numerical analyses employing the finite element approach within the ABAQUS^®^ software. The investigation spanned the entire load spectrum up to the point of structural failure, incorporating both practical trials and simulation analysis. During the practical assessments, the study monitored the post-buckling response and captured acoustic emissions to thoroughly evaluate the composite’s failure mechanisms. Additionally, the ARAMIS system’s non-invasive three-dimensional scanning was employed to assess deformations. Theoretical simulations utilized a step-by-step failure analysis, initiating with failure onset as per Hashin’s theory and proceeding to failure progression based on an energy criterion. The simulation outcomes, particularly concerning the critical and post-critical phases, were juxtaposed with empirical data to identify the composite’s vulnerability zones. The comparison underscored a significant concordance between the simulation predictions and the empirical findings.

## 1. Introduction

Thin-walled structures form a critical part of load-bearing frameworks utilized in modern engineering, including aerospace [1,2,3], automotive, and construction applications [4,5,6]. These structures are distinguished by their superior strength characteristics, which contribute to a high load-bearing capacity while maintaining a minimal self-weight. The increasing adoption of composite materials, susceptible to complex load conditions, necessitates in-depth investigations. Analytical methods and assessments of the stress levels in composite structures are continually being enhanced, particularly concerning their load-bearing capabilities. The nature of thin-walled load-bearing structures means that under certain load conditions, individual elements may risk stability loss within operationally permissible loads. Hence, beyond strength criteria, specific rigidity requirements are imposed to prevent early destruction due to stability loss in their elements, as discussed in various studies [7,8,9,10,11].

The buckling effects observed in thin-walled profiles are generally undesirable; numerous studies have demonstrated that stability loss in the form of elastic buckling, with a stable post-buckling equilibrium path, allows for the continued bearing of compressive loads in the post-buckling range [12,13,14].

In the critical phase, thin-walled structures made of composites often do not show any apparent signs of damage because the structure typically maintains its capability to support axial compressive forces even after surpassing the critical load. The functionality of these constructions is commonly linked with the likelihood of damage to the composite structure occurring predominantly in the extended post-critical operational phase [15,16]. The emergence of damage in this advanced post-critical stage can markedly diminish the mechanical integrity of the composite structure, thus posing a risk to its safe functionality even within the bounds of operationally acceptable loads. The multifaceted nature of damage and failure in thin-walled composite constructions is highlighted by a variety of phenomena that accompany irreversible structural damage, including the breakage of fibers or matrix and delamination [17,18,19,20,21,22,23,24].

The complexity of damage and destruction phenomena within composite material structures necessitates thorough analysis within ongoing research. Analyzing the full spectrum of loads initiates with a phase known as the damage initiation of the composite material, followed by a progression toward load-bearing capacity loss directly associated with damage evolution. The foundational theory for describing in detail the failure of thin-walled composite elements is known as the First Ply Failure theory (FPF) [25,26,27,28,29]. According to this theory, the composite is deemed to fail upon the damage state reaching the first layer of the laminate. This can be observed during experimental studies that utilize acoustic signal recording techniques (count numbers, hits numbers, signal amplitude, or energy) based on the acoustic emission method [30,31,32,33,34]. For numerical calculations, damage initiation assessment can be conducted using established damage initiation criteria, where Hashin’s damage initiation criterion is of particular significance [35]. This criterion assesses the damage initiation state based on reaching the initial damage parameters of the composite material, including fiber damage due to tensile and compression forces and matrix damage due to tensile and compression forces. Moreover, utilizing Hashin’s criterion [10,36,37,38] allows for further analysis within the subsequent damage evolution (with additional use of the energy criterion).

The preliminary concept posits that the damage can be conceptualized as the emergence of specific micro-fractures or the diminution of the effective cross-sectional area due to these micro-fractures, as introduced by Kachanov [39] in his delineation of destruction. This perspective envisages that the structure’s load is conveyed solely through its intact cross-sectional area. The prevalent discourse in the literature focuses on Progressive Damage Analysis (PDA) [40,41,42], with the onset of damage being identified based on Hashin’s criterion discussed earlier. PDA necessitates the specification of a suitable material model incorporating parameters for the initiation and progression of damage. The diminishment in stiffness following the onset of damage adheres to the framework put forth by Matzenmiller [43]. The gradual decrement in material stiffness is governed by variable parameters designed for the progression of damage. PDA identifies five key elements corresponding to the deterioration of material characteristics: damage under tensile and compressive forces in fibers, damage under tensile and compressive forces in the matrix, and damage from interlaminar shear [8,44].

This study provides a comprehensive experimental and numerical analysis of the buckling and subsequent post-buckling responses of compressed thin-walled composite plates, with a particular emphasis on asymmetric layouts. Additionally, this paper employs multidisciplinary research techniques that facilitate the examination of nonlinear stability problems in conjunction with the destruction phase of the composite material. This study meticulously documents the experimental process, which involves axial compression tests complemented by acoustic emission techniques to track damage evolution. Numerical simulations, employing finite element analysis (FEA) through ABAQUS^®^ 2024, reinforce the experimental findings by providing detailed insights into the critical and post-critical states of the structures. This dual approach not only confirms the experimental observations but also highlights the significance of progressive failure analysis in understanding the material and structural integrity under compressive load.

A novelty in terms of research activity was the use of asymmetric systems and the mechanical couplings occurring in them in order to obtain an element that can work as an elastic element. The research discussed herein stemmed from the necessity to understand the impact of mechanical coupling in plate elements functioning as elastic components. Engineering machines and devices occasionally demand the incorporation of protective elements with minimal weight and precise operational traits. Furthermore, designing machines often involves the integration of elastic components within confined, rectangular spaces. In both scenarios, plates featuring cut-outs prove to be a viable solution. Previous research works on plate elements [11,45] mainly covered the critical and low-critical states. Therefore, the novelty of this work is also the description of the phenomenon of destruction using interdisciplinary research methods.

## 2. Study Subject and Material Properties

**Figure 1:**
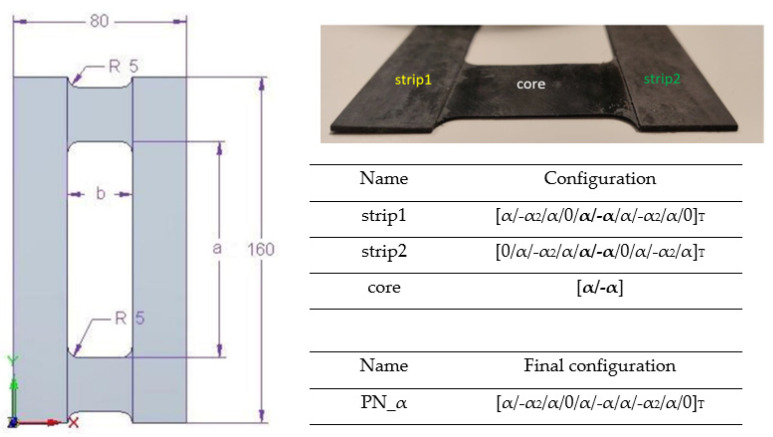
Geometry and layup configuration.

The focus of this research was on thin-walled plates made from carbon–epoxy composite material. The tested plates consisted of 12 layers in asymmetrical configuration arrangements relative to the laminate middle plane: [α/−α_2_/α/0/α/−α/α/−α_2_/α/0]_T_, where α means the angle of fiber arrangement and equaled 30°, 45°, and 60°. Each layer’s thickness was precisely 0.105 mm. Every tested plate element featured a central rectangular cut-out measuring 40 mm in width (b) and 100 mm in length (a). The plate geometry and composite configuration were informed by earlier studies [46,47,48]. Enhancements to the performance of slender plate elements and the assurance of their work within the elastic regime post-buckling were achieved through the introduction of a central cut-out and an asymmetrical layering technique. The determination of the layer arrangement stemmed from selecting suitable mechanical couplings from the B matrix, wherein the tested scenario involves strips and a core characterized by A_S_B_T_D_S_ mechanical couplings. The methodology for choosing mechanical couplings has been elaborated further in several studies [46,49,50,51]. This method led to the creation of a novel elastic component concept. Such a strategy facilitated the bending of the plate’s vertical segments in reverse directions during the compression tests [46]. The geometry and layup configurations of samples are presented in Figure 1. The tested samples were manufactured using the autoclave method under special and sterile conditions. This method facilitated the creation of a composite structure featuring a unique fiber content percentage within the range of 55–60%.

The material properties were experimentally established in accordance with ISO standards: the compression test followed PN-EN ISO 14126 (from 2002) [52], the static tensile test adhered to PN-EN ISO 527–5 (from 2010) [53], and the shear test complied with PN-EN ISO 14129 (from 2000) [54]. Details on the procedure for determining material properties are outlined in [55]. The material properties are presented in Table 1.

## 3. Experimental Test

**Figure 2:**
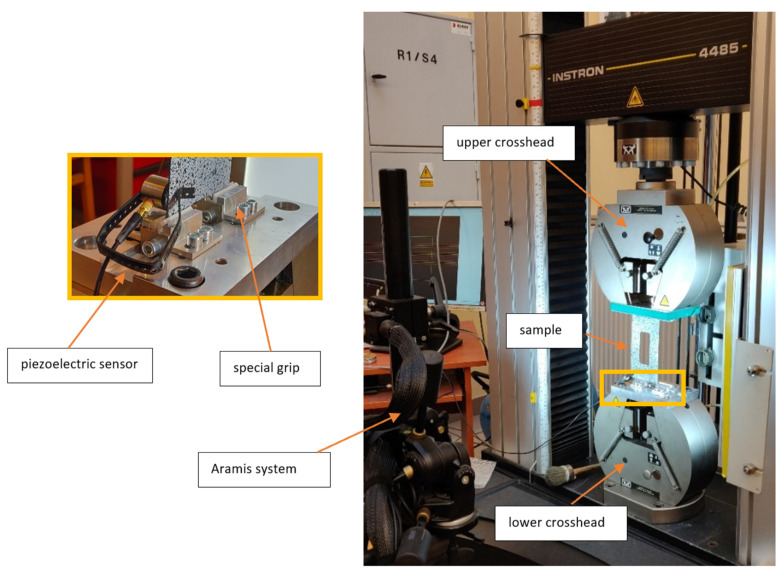
Experiment test stand for compression tests.

Thin-walled CFRP (Carbon Fiber-Reinforced Polymer) plates underwent compressive damage testing. The experiments were conducted using the Instron universal testing machine under ambient conditions at a temperature of 23 °C and a constant crosshead speed of 2 mm/min. The testing apparatus was specially outfitted with designed grips on both the upper and lower crossheads to ensure the samples received articulated support (refer to Figure 2). The grips had cylindrical inserts with notches where the plate could be mounted. The inserts were made of Teflon material, which ensured a low friction coefficient during the tests. This setup closely aligned with the freely supported boundary conditions assumed in the numerical analysis [56]. Testing on physical models of thin-walled elements, which were fabricated using the autoclave technique [57,58,59], facilitated the validation of the numerical model.

Experimental tests were conducted on composite samples, with the laminate ply configuration as previously described, at three distinct fiber orientations: 30°, 45°, and 60°. The tests measured several parameters: the compressive force applied, the shortening of the plate, the deflection in the direction perpendicular to the plate surface (notably at the mid-height of the plate’s vertical strips, where deflection peaked), and the acoustic emission signals. For measuring deflection, ARAMIS by GOM was utilized [60,61], offering comprehensive visualization of displacements and strains. Acoustic emission signals [30] were captured using an AMSY-5 system equipped with a Fujicera 1045S piezoelectric sensor and an AEP-4 signal amplifier, allowing for the documentation of the initial damage indicators in terms of energy, amplitude, counts, and sum of counts. The experimental outcomes were then juxtaposed with the plate shortening predictions derived from numerical simulations (FEM). The author has elaborated on the experimental procedures in earlier publications [62,63].

In the course of the experimental tests, two critical forces were identified: the force initiating damage in the first composite layer (P_d_), and the failure force (P_f_), which corresponds to the point at which the structure loses its load-bearing capacity.

## 4. Numerical Analysis

The numerical analyses conducted in Abaqus software 2024 are grounded in the Finite Element Method (FEM), renowned for its extensive applicability [2,64,65,66,67,68,69,70]. This analysis of compressed composite plates unfolded in two distinct phases. Initially, a linear eigenproblem was addressed based on the minimum potential energy principle [71] Subsequently, the second phase of numerical analysis involved conducting nonlinear stability assessments employing the Newton–Raphson incremental–iterative method [72,73,74]. These assessments accounted for geometric imperfections aligning with the flexural–torsional buckling mode. The research utilized six-node shell elements, each endowed with six degrees of freedom. The geometric imperfection used in the analysis equaled 0.1 of the plate thickness. Abaqus software’s section configuration capabilities facilitated the distinct definition of each laminate ply, including material properties, thickness, and the principal axes of the orthotropic material. Material parameters are detailed in Table 1, where the properties of the composite material were characterized by an orthotropic material model under plane stress conditions.

**Figure 3:**
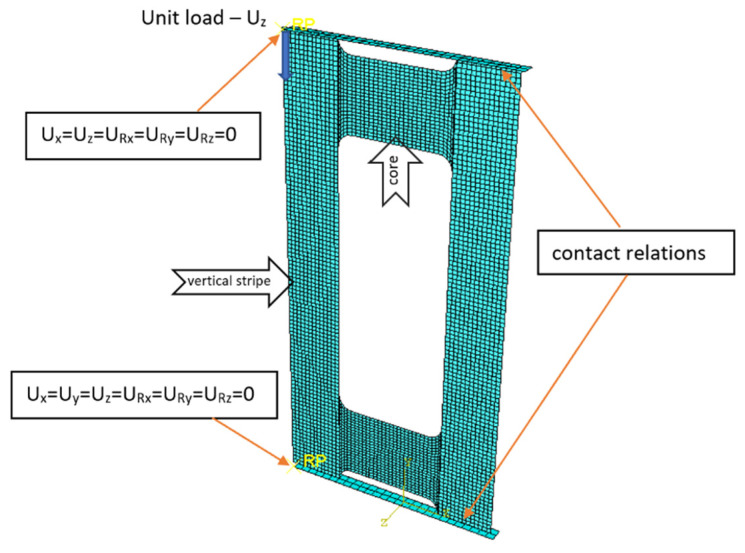
FE model with boundary conditions.

The plate elements’ geometry was meticulously crafted to mirror the dimensions of the actual specimens, as depicted in Figure 1. To accurately replicate the experimental test conditions, two rigid plates were simulated to serve as the top and bottom edge supports for the test plate. These support plates were modeled using rigid shell elements of the R3D4 type, each endowed with three translational degrees of freedom at its four computational nodes within each finite element. Reference points were established in conjunction with these non-deformable plates, where the boundary conditions were specified (see Figure 3). The constructed numerical model comprised 4244 finite elements, ensuring a detailed representation of the physical testing setup. The element size of 1.5 mm was used in subsequent analysis. Mesh convergence analysis was performed in previous papers [75].

Numerical analyses were carried out across the entire spectrum of loads, culminating in total failure, employing Progressive Failure Analysis (PFA) grounded in the Hashin failure criterion and an energy criterion pertinent to damage evolution [8,35,44,76]. This criterion enables the evaluation of damage levels within the composite material attributable to fiber tension (HSNFTCRT), fiber compression (HSNFCCRT), matrix tension (HSNMTCRT), and matrix compression (HSNMCCRT). When any of these damage initiation criteria meet or exceed a value of 1 for the failure initiation parameter, it indicates the potential for further damage progression within a specific material component via PFA, while the rest of the components remain intact.

In line with Hashin’s theory, the activation of a damage initiation criterion for any component of the composite triggers a gradual reduction in that component’s stiffness, leading eventually to a total loss of material stiffness. The damage evolution model incorporates five independent failure parameters for the composite material, each corresponding to failure modes such as fiber compression (DAMAGEFC), fiber tension (DAMAGEFT), matrix compression (DAMAGEMC), matrix tension (DAMAGEMT), and interlaminar shear (DAMAGESHR).

Further details on the methodologies applied can be found in the referenced literature [62,63,75,77,78].

## 5. Results and Discussion

**Figure 4:**
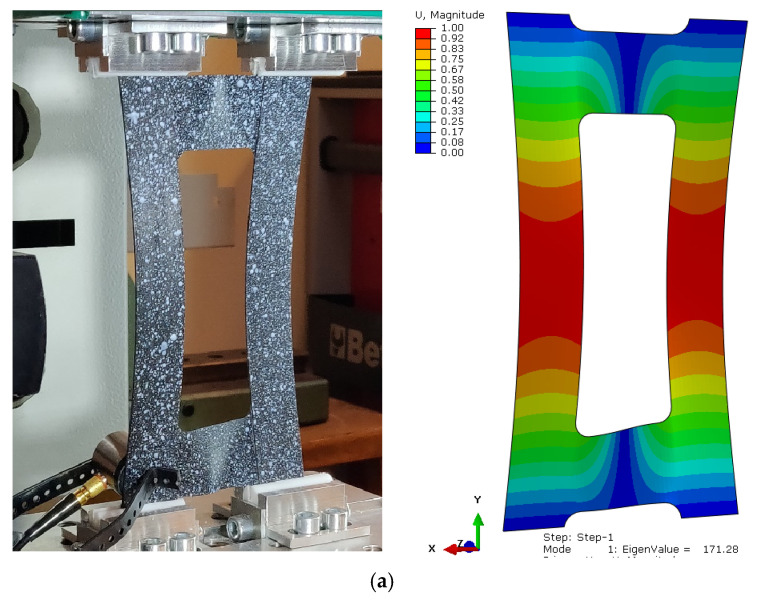

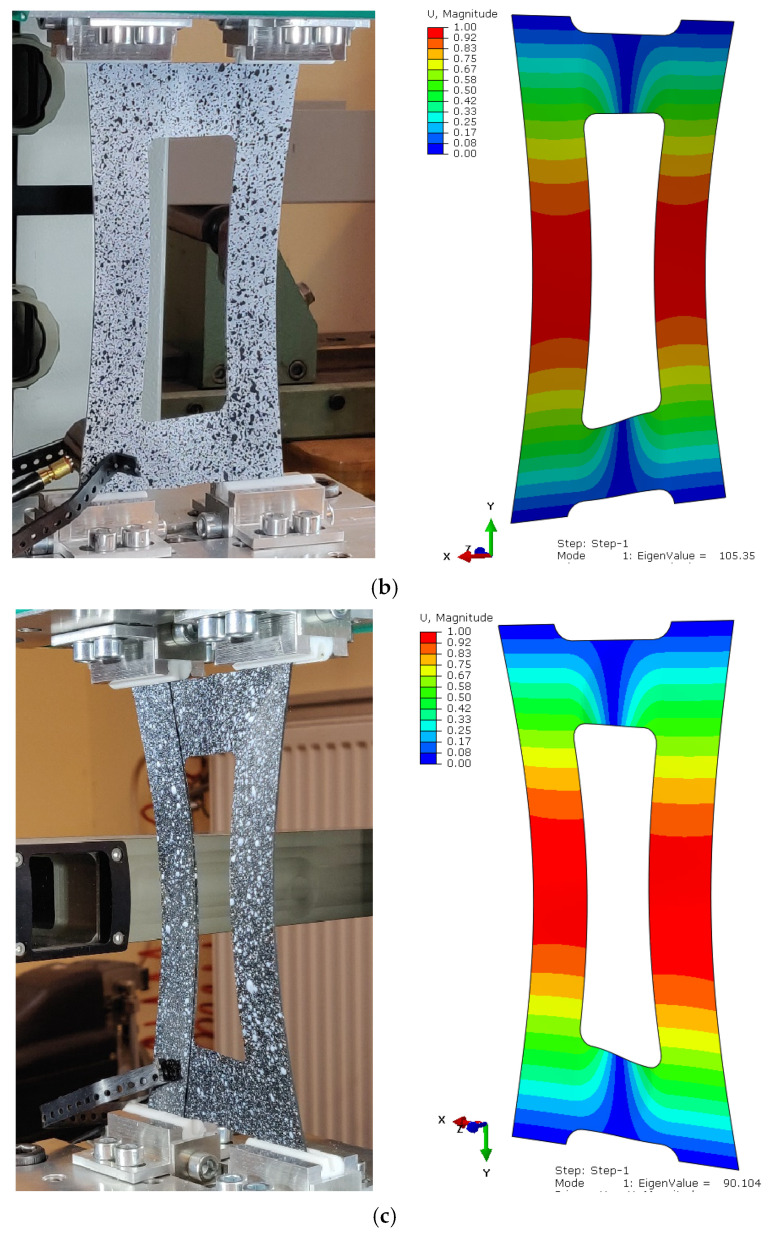
Experimental and numerical buckling form: PN_30 (**a**); PN_45 (**b**); PN_60 (**c**).

The investigation into the buckling behaviors of compressed composite plates with central cut-outs, through both experimental and numerical approaches, revealed a qualitative concurrence. Moreover, the analytical calculations were used to indicate A, B, and D matrix values to confirm a coupling effect [79,80,81]. Experimental analyses verified that the primary mode of buckling was indeed flexural–torsional, demonstrating stability within the post-buckling range. Moreover, the analysis of the critical state, via numerical calculations, facilitated the identification of various buckling forms and the corresponding critical loads for each specific layer configuration within the composite material, as illustrated in Figure 4. Such observations affirm the efficacy of the chosen asymmetric fiber arrangement and the mechanical couplings. Equations (1)–(3) present the results of analytical calculations of constitutive relations for considered configurations. The calculations were performed using the Matlab program.

The ABD matrix for the PN_30, PN_45, and PN_60 configuration is given in Equations (1)–(3), respectively, and represents E-B-S-B-E-T-S-T-T-B coupling.

(1) $$\left\{\begin{array}{c} \left\{\begin{array}{c} N_{x}\\ N_{y}\\ \begin{array}{c} N_{xy}\\ \ldots \end{array} \end{array}\right\}\\ \left\{\begin{array}{c} M_{x}\\ M_{y}\\ M_{xy} \end{array}\right\} \end{array}\right\}=\left[\begin{array}{cc} \begin{array}{ccc} 119763.91 & 28358.47 & 0\\ 28358.47 & 18016.22 & 0\\ \begin{array}{c} 0\\ \cdots \end{array} & \begin{array}{c} 0\\ \cdots \end{array} & \begin{array}{c} 30546.52\\ \cdots \end{array} \end{array} & \begin{array}{ccc} \begin{array}{cc} \vdots \;\;\; & \;2605.69\;\; \end{array}\; & -1079.48 & -486.24\\ \begin{array}{cc} \vdots \; & -1079.48 \end{array}\;\;\; & -446.74 & -174.63\\ \begin{array}{c} \begin{array}{cc} \vdots & \;\;-486.24 \end{array}\;\;\;\\ \;\begin{array}{cc} \cdots & \;\;\;\;\;\cdots \end{array}\;\;\;\;\;\;\;\;\;\;\;\;\; \end{array} & \begin{array}{c} \;-174.63\\ \cdots \end{array} & \begin{array}{c} -1079.48\\ \cdots \end{array} \end{array}\\ symmetry & \begin{array}{ccc} \begin{array}{cc} \vdots & \;\;\;\;\;16437.56 \end{array} & \;\;3506.25 & \;\;\;\;\;\;408.45\\ \begin{array}{cc} \vdots \;\;\;\;\;\;\;\; & 3506.25 \end{array} & \;2281.91 & \;\;\;\;\;\;146.69\\ \begin{array}{cc} \vdots \;\;\;\;\;\; & \;408.45 \end{array}\; & \;\;\;146.69 & \;\;\;\;\;3795.72 \end{array} \end{array}\right]=\begin{array}{c} \left\{\begin{array}{c} \varepsilon_{x}\\ \varepsilon_{y}\\ \begin{array}{c} \gamma_{xy}\\ \ldots \end{array} \end{array}\right\}\\ \left\{\begin{array}{c} {𝒦}_{x}\\ {𝒦}_{y}\\ {𝒦}_{xy} \end{array}\right\} \end{array}$$

(2) $$\left\{\begin{array}{c} \left\{\begin{array}{c} N_{x}\\ N_{y}\\ \begin{array}{c} N_{xy}\\ \ldots \end{array} \end{array}\right\}\\ \left\{\begin{array}{c} M_{x}\\ M_{y}\\ M_{xy} \end{array}\right\} \end{array}\right\}=\left[\begin{array}{cc} \begin{array}{ccc} 74858.17 & 36925.75 & 0\\ 36925.75 & 45787.4 & 0\\ \begin{array}{c} 0\\ \cdots \end{array} & \begin{array}{c} 0\\ \cdots \end{array} & \begin{array}{c} 39113.8\\ \cdots \end{array} \end{array} & \begin{array}{ccc} \begin{array}{cc} \vdots \;\;\; & \;4491.73\;\; \end{array}\; & -1439.3 & -381.55\\ \begin{array}{cc} \vdots \; & -1439.3 \end{array}\;\;\; & -1613.13 & -381.55\\ \begin{array}{c} \begin{array}{cc} \vdots & \;\;-381.55 \end{array}\;\;\;\\ \;\begin{array}{cc} \cdots & \;\;\;\;\;\cdots \end{array}\;\;\;\;\;\;\;\;\;\;\;\;\; \end{array} & \begin{array}{c} \;-381.55\\ \cdots \end{array} & \begin{array}{c} -1439.3\\ \cdots \end{array} \end{array}\\ symmetry & \begin{array}{ccc} \begin{array}{cc} \vdots & \;\;\;\;\;10925.61 \end{array} & \;\;4557.84 & \;\;\;\;\;\;320.51\\ \begin{array}{cc} \vdots \;\;\;\;\;\;\;\; & 4557.84 \end{array} & \;5690.69 & \;\;\;\;\;\;320.51\\ \begin{array}{cc} \vdots \;\;\;\;\;\; & \;320.51 \end{array}\; & \;\;\;320.51 & \;\;\;\;\;4847.31 \end{array} \end{array}\right]=\begin{array}{c} \left\{\begin{array}{c} \varepsilon_{x}\\ \varepsilon_{y}\\ \begin{array}{c} \gamma_{xy}\\ \ldots \end{array} \end{array}\right\}\\ \left\{\begin{array}{c} {𝒦}_{x}\\ {𝒦}_{y}\\ {𝒦}_{xy} \end{array}\right\} \end{array}$$

(3) $$\left\{\begin{array}{c} \left\{\begin{array}{c} N_{x}\\ N_{y}\\ \begin{array}{c} N_{xy}\\ \ldots \end{array} \end{array}\right\}\\ \left\{\begin{array}{c} M_{x}\\ M_{y}\\ M_{xy} \end{array}\right\} \end{array}\right\}=\left[\begin{array}{cc} \begin{array}{ccc} 47086.99 & 28358.47 & 0\\ 28358.47 & 90693.14 & 0\\ \begin{array}{c} 0\\ \cdots \end{array} & \begin{array}{c} 0\\ \cdots \end{array} & \begin{array}{c} 30546.52\\ \cdots \end{array} \end{array} & \begin{array}{ccc} \begin{array}{cc} \vdots \;\;\; & \;5658.12\;\; \end{array}\; & -1079.48 & -174.63\\ \begin{array}{cc} \vdots \; & -1079.48 \end{array}\;\;\; & -3499.17 & -486.24\\ \begin{array}{c} \begin{array}{cc} \vdots & \;\;-174.63 \end{array}\;\;\;\\ \;\begin{array}{cc} \cdots & \;\;\;\;\;\cdots \end{array}\;\;\;\;\;\;\;\;\;\;\;\;\; \end{array} & \begin{array}{c} \;-486.24\\ \cdots \end{array} & \begin{array}{c} -1079.48\\ \cdots \end{array} \end{array}\\ symmetry & \begin{array}{ccc} \begin{array}{cc} \vdots & \;\;\;\;\;7516.83 \end{array} & \;\;3506.25 & \;\;\;\;\;\;146.69\\ \begin{array}{cc} \vdots \;\;\;\;\;\;\;\; & 3506.25 \end{array} & \;11202.64 & \;\;\;\;\;\;408.45\\ \begin{array}{cc} \vdots \;\;\;\;\;\; & \;146.69 \end{array}\; & \;\;\;408.45 & \;\;\;\;\;3795.72 \end{array} \end{array}\right]=\begin{array}{c} \left\{\begin{array}{c} \varepsilon_{x}\\ \varepsilon_{y}\\ \begin{array}{c} \gamma_{xy}\\ \ldots \end{array} \end{array}\right\}\\ \left\{\begin{array}{c} {𝒦}_{x}\\ {𝒦}_{y}\\ {𝒦}_{xy} \end{array}\right\} \end{array}$$

**Figure 5:**
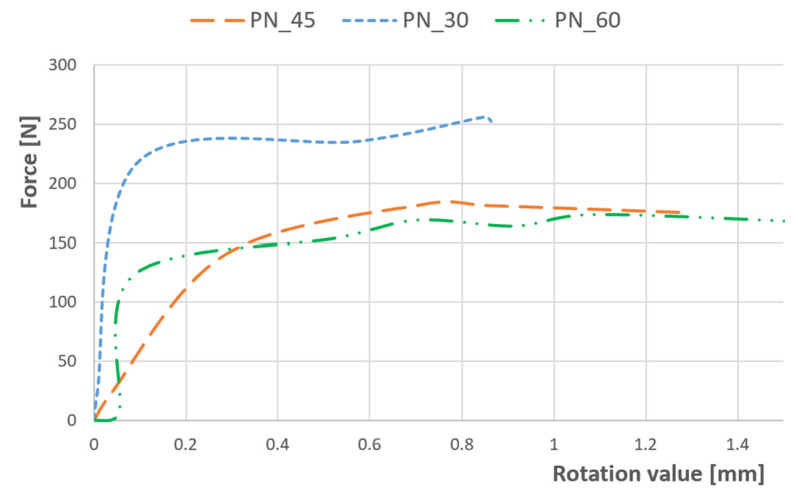
Results for compression load vs. rotation value for three different angles.

The considered configurations indicate developed *Bending–Twisting* behaviour (B_16_, B_26_ ≠ 0), and the addition of *Bending–Twisting* coupling stiffnesses D_16_ and D_26_ in laminate causes *Twisting* because of *Bending*. Moreover, Figure 5 presents the relations between compression force and the rotation value, measurements for the plate strip on one side and on the other side.

The rotational value shows a notable increase, particularly when the load approaches the buckling threshold. Additionally, there is a discernible rise in the twisting response of the specimen upon the introduction of laminate coupling effects. The results obtained provide clear evidence of coupling interactions occurring, which contribute to the amplified twisting response.

**Figure 6:**
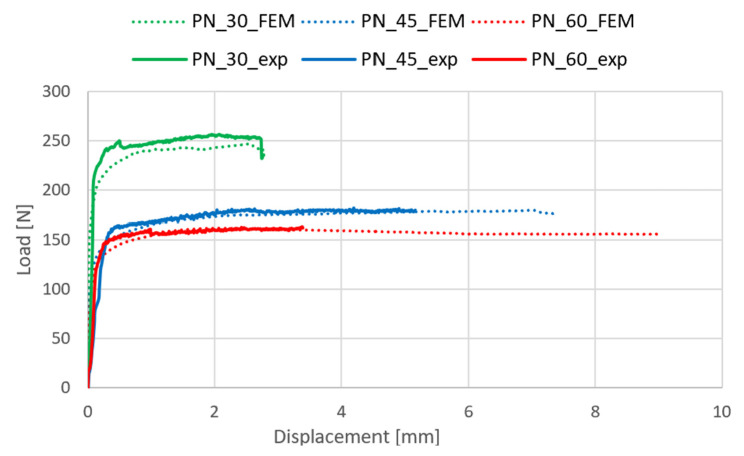
Comparison of experimental and numerical post-buckling equilibrium paths.

**Figure 7:**
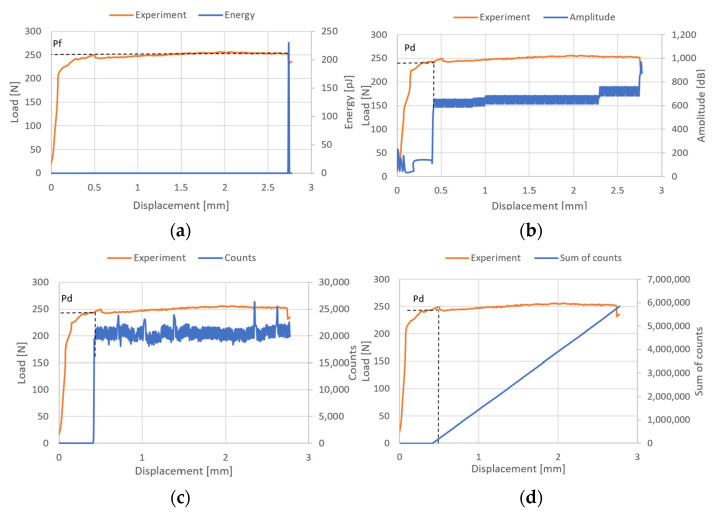
Post-critical equilibrium paths with acoustic emission signals for PN_30: (**a**) energy; (**b**) amplitude; (**c**) counts; (**d**) sum of counts.

**Figure 8:**
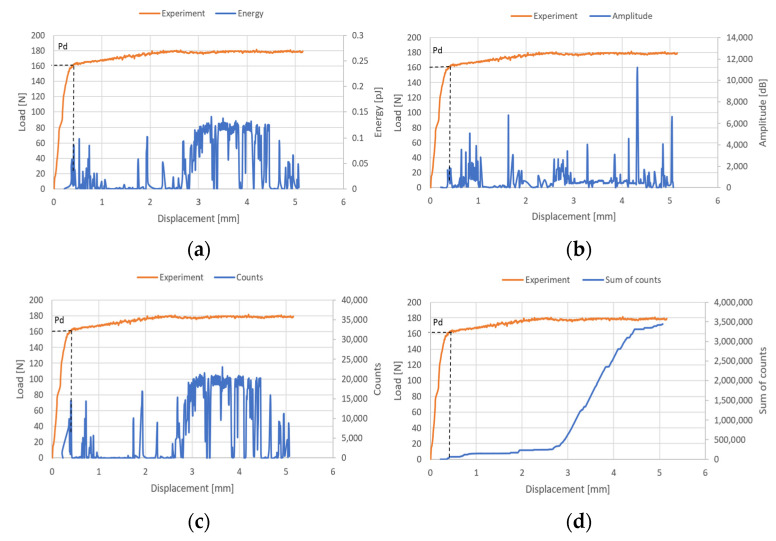
Post-critical equilibrium paths with acoustic emission signals for PN_45: (**a**) energy; (**b**) amplitude; (**c**) counts; (**d**) sum of counts.

**Figure 9:**
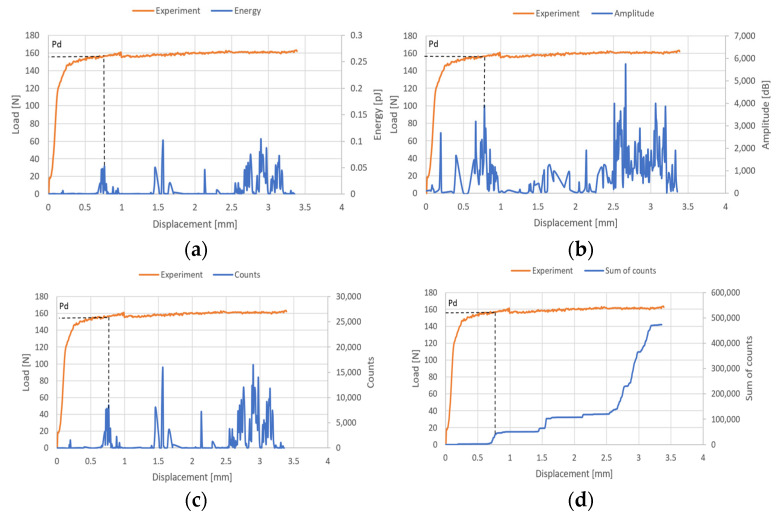
Post-critical equilibrium paths with acoustic emission signals for PN_60: (**a**) energy; (**b**) amplitude; (**c**) counts; (**d**) sum of counts.

The examination of the structure’s response to compression enabled the identification of post-critical working paths (compressive load versus displacement/shortening). The analysis revealed that these post-critical equilibrium paths exhibit a stable nature, indicating that an increase in the shortening of the thin-walled structure is paralleled by a rise in compressive load, even in the face of stability loss—as illustrated in Figure 6. Within the post-critical domain, continuous loading of the structure led to a phase termed damage initiation. During the experimental phase, parameters of the acoustic emission signal, including the number of counts, energy, and amplitudes, were meticulously recorded. These data subsequently facilitated the evaluation of the onset of damage. The results obtained are shown in Figure 7, Figure 8 and Figure 9. It is noteworthy that experimentally, only sample PN_30 succeeded in bringing the sample to complete failure.

**Figure 10:**
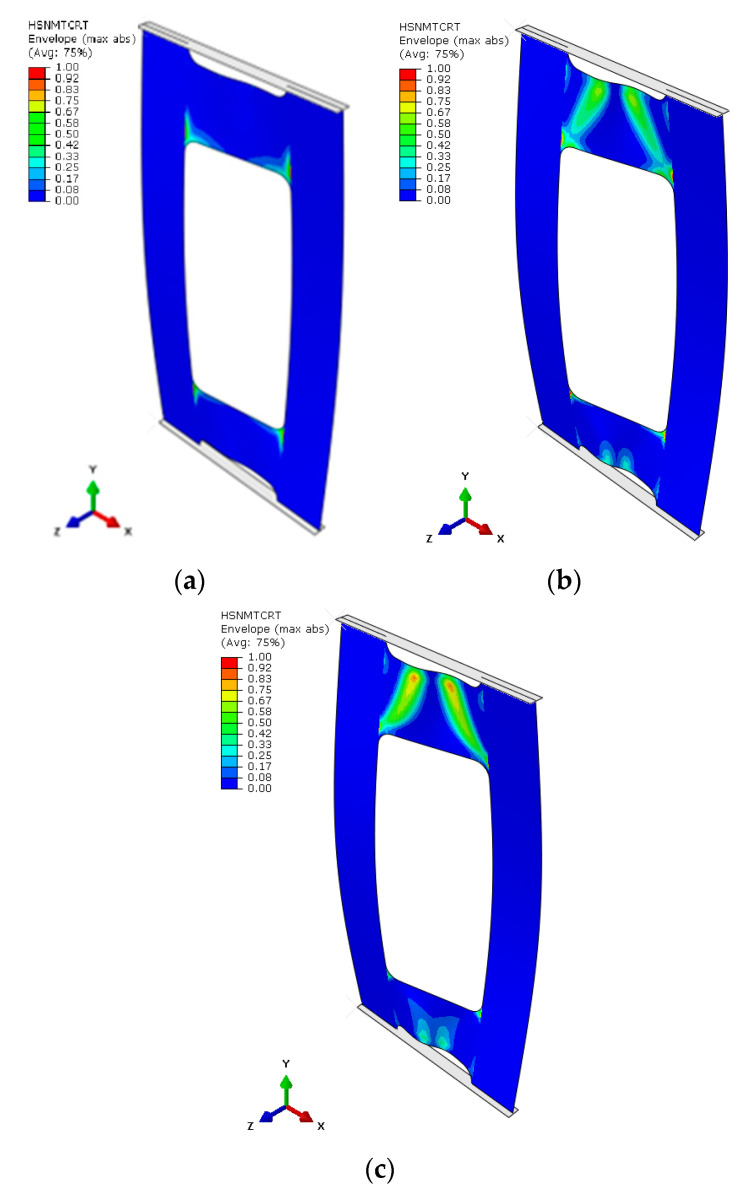
Damage initiation of plate (numerical analysis): PN_30 (**a**); PN_45 (**b**); PN_60 (**c**).

Based on the results obtained, it turned out that the sum of counts was the clearest acoustic emission signal in assessing the initiation of damage. On the other hand, for the loss of load-bearing capacity of the structure, thanks to the occurrence of a characteristic “peak” of energy, the energy parameter can also be recognized. However, it could only be recorded for specimen PN_30, as the other specimens failed to initiate damage. Upon examining the amplitude charts, it becomes evident that the amplitude of the acoustic emission signals exhibits a trend of increment in their recorded values as the duration of the measurement extends. This observation signifies that, with the progression of the testing period, the acoustic emissions, as quantified by their amplitudes, intensify. Such an escalation is typically indicative of an accumulation of damage within the material or structure under evaluation. By capturing these signals, the acoustic emission technique serves as a sensitive method to detect early and developing damages, like micro-cracking or the fracturing of fibers, within composite materials subjected to load. The increasing amplitudes imply that the material encounters more significant stress or damage events as the testing proceeds, offering valuable insights into the behavior of the material under sustained load conditions. It is worth noting that for samples PN_45 and PN_60, based on the results of a single signal, it would be difficult to unambiguously determine the value of the load initiating composite failure (P_d_) compared to sample PN_30. It can be observed that P_d_ damage initiation for all samples occurred due to matrix tension—HSNMTCRT (Figure 10). Figure 10 presents the maps showing the level of damage evolution after the fulfillment of at least two components (PN_30:DAMAGEMT, DAMAGESHR; PN_45:DAMAGEMT, DAMAGEMC, DAMAGESHR; PN_60:DAMAGEMT, DAMAGEMC, DAMAGESHR, DAMAGEFC). The presented results confirm the influence of the fiber arrangement on both the value of the critical force and the initiation and failure evolution. Additionally, it is related to the influence of mechanical couplings in the chosen configuration. However, this work mainly focused on the stability and failure of thin-walled composite plate elements as well as the angle of the fiber arrangement.

**Figure 11:**
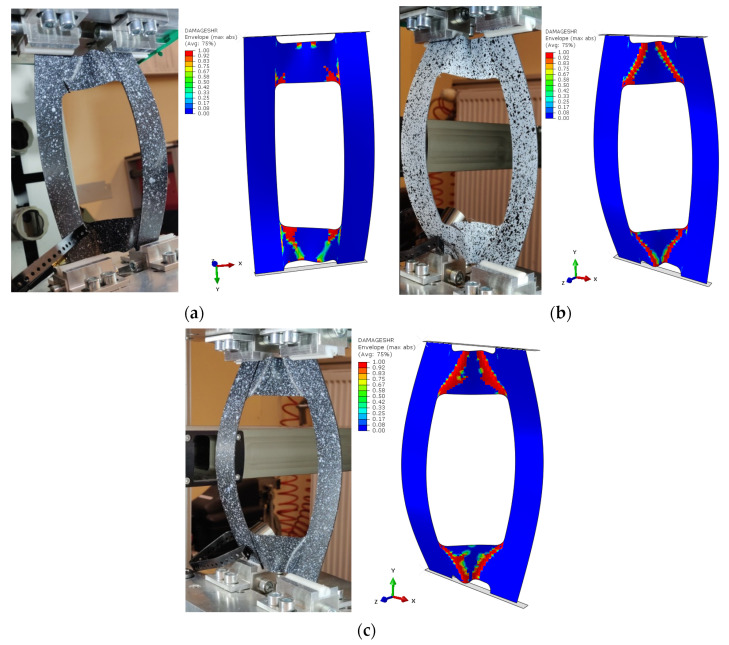
Comparison of the damage evolution results from experimental test and FEM analysis: PN_30 (**a**); PN_45 (**b**); PN_60 (**c**).

**Figure 12:**
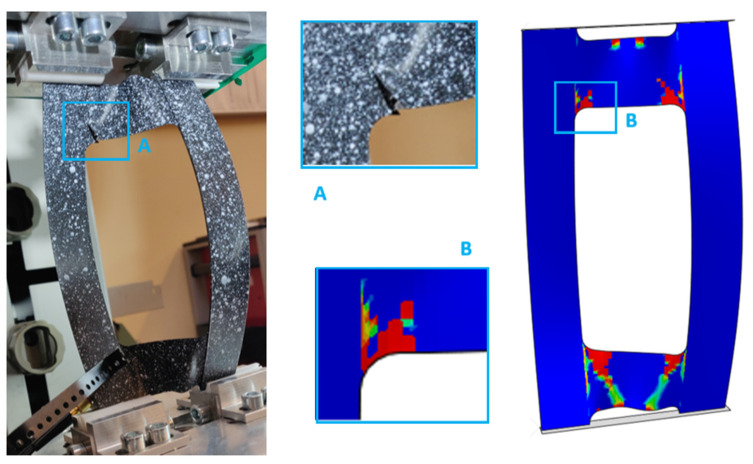
Graphical presentation of the form of failure of the plate in PN_30 configuration.

The area of damage observed in the experimental research aligns with the area predicted by the numerical simulations (Figure 11). In every scenario, the zones near the cut-out corners, as well as the core area, emerged as the most prone to damage, with destruction primarily initiated at the corners. A pattern was discernible, indicating that an increase in the fiber alignment angle tends to decrease the plate’s rigidity, leading to stress concentration in the core. As previously noted, failure was only achieved in the case of the PN_30 plate. According to Figure 12, the material failure that compromised the plate’s load-bearing capacity was located near the upper left corner of the cut-out and in the section of the core linking to the plate’s vertical strip.

To provide a quantitative summary of the test outcomes across the three distinct fiber orientation configurations, the collected results have been compiled and are showcased in Table 2. In addition, the last columns include the value of the relative error with respect to the experimental values. 

The limit state analysis showed that the relative error between experimental and numerical tests for the load initiating damage of the first composite layer was 5.65% for PN_30, 0.49% for PN_45, and 2.05% for PN_60. On the other hand, in the case of the load initiating delamination and the load causing a loss of load capacity for PN_30, it was 4.98%.

The results obtained allow for the evaluation of the accuracy in the development of the numerical model, which was corroborated by the outcomes of the experimental tests.

## 6. Conclusions

This paper presents the results of comprehensive experimental and numerical investigations into the stability and failure of thin-walled composite plates subjected to axial compression, focusing on asymmetric layer configurations. This research utilized carbon–epoxy composite materials crafted through the autoclave technique, meticulously exploring the entire load spectrum up to structural failure. By integrating experimental tests with numerical simulations via the finite element method in ABAQUS^®^, this study illuminated the complex failure mechanisms of the composites. Particularly, the research leveraged acoustic emission monitoring and the ARAMIS non-invasive scanning system for deformation assessment, alongside a progressive failure analysis based on Hashin’s criterion and an energy criterion for detailed failure evolution mapping.

Key findings from this investigation include the validation of flexural–torsional buckling as the primary mode of failure, which was consistently observed in both experimental and numerical analyses. This buckling mode’s identification underscores the effectiveness of the selected asymmetric fiber layout and mechanical couplings in ensuring the structure’s stability beyond the critical load. Additionally, this study identified the critical and failure loads for various layer configurations, enhancing the understanding of how composite layer arrangements influence structural integrity.

A notable innovation in this research was the application of asymmetric layouts and the exploration of mechanical couplings within these structures to create elements capable of functioning as elastic components. This approach, which deviates from traditional studies focusing primarily on critical and low-critical states, offers a novel perspective on the destruction phenomenon using interdisciplinary methods.

In summary, this work contributes significantly to the field of composite structure analysis by providing a detailed examination of the buckling behavior and failure mechanisms of thin-walled composite plates with asymmetric configurations. The synergy between experimental insights and numerical predictions offers a robust framework for predicting composite structures’ behavior under axial compression, paving the way for designing more resilient composite materials and structures in the future. This research was made possible by funding from the National Science Centre Poland, underlining its scientific significance and its impact on advancing composite materials research.

## Figures and Tables

**Table 1:** Mechanical and strength properties of composite material.

Mechanical Properties				
Young’s modulus E_1_ [MPa]	Young’s modulus E_2_ [MPa]	Poisson’s ratio [-]		Kirchhoff modulus G_12_ [MPa]
143,530	5826	0.36		3845
**Strength Properties**				
Tensile strength (0°) F_T1_ [MPa]	Compressive strength (0°) F_C1_ [MPa]	Tensile strength (90°) F_T2_ [MPa]	Compressive strength (90°) F_C2_ [MPa]	Shear strength F_12_ [MPa]
2221	641	49	114	83.5

**Table 2:** Limit load results.

	Experimental Test [N]			FEM Analysis [N]			Error [%]		
	PN_30	PN_45	PN_60	PN_30	PN_45	PN_60	PN_30	PN_45	PN_60
Damage initiation	242	160.31	156.87	228.31	161.09	160.09	5.65	0.49	2.05
Failure	251.87	-	-	239.33	178.34	-	4.98	-	-

## Data Availability

Data are contained within the article.
